# Cuproptosis-related gene signature stratifies lower-grade glioma patients and predicts immune characteristics

**DOI:** 10.3389/fgene.2022.1036460

**Published:** 2022-10-25

**Authors:** Zihao Zhang, Bingcheng Wang, Xiaoqin Xu, Tao Xin

**Affiliations:** ^1^ Department of Surgery, Shandong Provincial Hospital, Shandong University, Jinan, China; ^2^ Department of Neurosurgery, Shandong Provincial Qianfoshan Hospital, Shandong University, Jinan, China; ^3^ Shandong Medicine and Health Key Laboratory of Neurosurgery, The First Affiliated Hospital of Shandong First Medical University and Shandong Provincial Qianfoshan Hospital, Jinan, China; ^4^ Department of Endocrinology, Shandong Provincial Hospital, Shandong University, Jinan, China; ^5^ Department of Neurosurgery, Jiangxi Provincial People’s Hospital Affiliated to Nanchang University, Nanchang, China

**Keywords:** lower-grade glioma, cuproptosis-related gene, gene signature, prognosis, immune score

## Abstract

Cuproptosis is the most recently discovered type of regulated cell death and is mediated by copper ions. Studies show that cuproptosis plays a significant role in cancer development and progression. Lower-grade gliomas (LGGs) are slow-growing brain tumors. The majority of LGGs progress to high-grade glioma, which makes it difficult to predict the prognosis. However, the prognostic value of cuproptosis-related genes (CRGs) in LGG needs to be further explored. mRNA expression profiles and clinical data of LGG patients were collected from public sources for this study. Univariate Cox regression analysis and the least absolute shrinkage and selection operator (LASSO) Cox regression model were used to build a multigene signature that could divide patients into different risk groups. The differences in clinical pathological characteristics, immune infiltration characteristics, and mutation status were evaluated in risk subgroups. In addition, drug sensitivity and immune checkpoint scores were estimated in risk subgroups to provide LGG patients with precision medication. We found that all CRGs were differentially expressed in LGG and normal tissues. Patients were divided into high- and low-risk groups based on the risk score of the CRG signature. Patients in the high-risk group had a considerably lower overall survival rate than those in the low-risk group. According to functional analysis, pathways related to the immune system were enriched, and the immune state differed across the two risk groups. Immune characteristic analysis showed that the immune cell proportion and immune scores were different in the different groups. High-risk group was characterized by low sensitivity to chemotherapy but high sensitivity to immune checkpoint inhibitors. The current study revealed that the novel CRG signature was related to the prognosis, clinicopathological features, immune characteristics, and treatment perference of LGG.

## Introduction

Worldwide, the incidence of central nervous system (CNS) tumors is 1.6%, while their mortality rate has reached 2.5% (Sung et al., 2021). According to the 2007 World Health Organization (WHO) classification of CNS tumors, gliomas are divided into four classes (i.e., WHO grades I, II, III, and IV) (Fan et al., 2016). Grade II and III gliomas are referred to as “diffuse lower-grade gliomas” (LGGs), which tend to have a better prognosis than higher grade gliomas. LGGs, which include astrocytomas, oligodendrogliomas, and oligoastrocytomas, are infiltrative neoplasms that account for 6.4% of CNS tumors (Brat et al., 2015). The primary treatment for LGG patients is surgery, radiotherapy and chemotherapy to relieve symptoms and improve the quality of life (Youssef and Miller, 2020). However, LGG relapse and progression can occur, usually owing to incomplete elimination of cancer cells, resulting in a broad range of overall survival times from 1 to 15 years, which makes prognosis prediction difficult (Smoll et al., 2012). Therefore, there is an urgent need to develop an innovative prognostic system.

Different types of regulated cell death, such as apoptosis, pyroptosis, necroptosis, and ferroptosis, have attracted much attention, as inducing regulated cell death is regarded as a novel approach for treating cancer patients (Lei et al., 2022). As new kinds of regulated cell death have been identified, our understanding of regulated cell death in cancer is continually expanding. Copper-dependent cell death, known as cuproptosis, is a type of regulated cell death that is induced by excessive copper ions (Hasinoff et al., 2014). According to recent research, cuproptosis triggers cell death by regulating the lipoylated components in the tricarboxylic acid (TCA) cycle, resulting in toxic protein stress. Furthermore, genes related to cuproptosis were identified, which are of great value to study (Tsvetkov et al., 2022). Many studies have proven that cuproptosis inhibits tumor growth and progression in colorectal cancer, hematopoietic cancers, and glioblastoma (Chen et al., 2019; Buccarelli et al., 2021; Gao et al., 2021). However, whether these cuproptosis-related genes (CRGs) are correlated with LGG patient prognosis remains to be further explored.

In the present study, the mRNA expression profiles and corresponding clinical data of LGG patients were downloaded from public databases. Then, a prognostic multigene signature was constructed based on CRGs in the TCGA cohort and validated in the CGGA cohort. This CRG signature divided LGG patients into high-risk and low-risk groups and predicted overall survival with high sensitivity and specificity. Gene Ontology (GO) and Kyoto Encyclopedia of Genes and Genomes (KEGG) functional enrichment analysis and Gene Set Enrichment Analysis (GSEA) were performed to determine the biological differences between patients in the high- and low-risk groups. Next, immune characteristics analyses, such as ssGSEA, CIBERSORT, and ESTIMATE, were performed to evaluate the tumor immune microenvironment differences between groups. Furthermore, the sensitivity to anticancer drugs and immune checkpoint inhibitors was predicted for patients in different risk groups. We discovered that differences in immune function and immune cell infiltration between the high-risk and low-risk groups were underlying features related to the ability of the gene signature to predict LGG patient prognosis.

## Materials and methods

### Data collection

RNA sequencing data were downloaded from The Cancer Genome Atlas (TCGA; https://portal.gdc.cancer.gov/) and Chinese Glioma Genome Atlas (CGGA; http://www.cgga.org.cn) databases (Liu et al., 2018). The gene expression profiles were normalized using the R package “limma.” Ten cuproptosis-related genes were identified from previous literature (Tsvetkov et al., 2022). A total of 182 samples were collected from the CGGA cohort, among which 172 samples with complete survival data were used to further validate the results from the TCGA cohort and were subjected to futher immune characteristic analysis.

### Construction and validation of a prognostic cuproptosis-related gene signature

In the TCGA cohort, the “DESeq2” R package was used to screen differentially expressed genes (DEGs) across tumor tissues and normal tissues with a false discovery rate (FDR) of 0.05. To identify cuproptosis-related genes with prognostic significance, a univariate Cox analysis of overall survival (OS) was performed. Benjamini and Hochberg (BH) adjustment was used to modify *p* values. The STRING database (version 11.0) was used to create an interaction network for the overlapping prognostic DEGs (Szklarczyk et al., 2011). To reduce overfitting, a CRG signature was built using LASSO-penalized Cox regression analysis (Tibshirani, 1997; Simon et al., 2011). With the “glmnet” R package, the LASSO algorithm was employed for variable selection and shrinkage. The normalized expression matrix of putative prognostic DEGs was used as the independent variable in the regression, while the response variables were overall survival and patient status in the TCGA cohort. The penalty parameter (λ) of the CRG signature was calculated *via* tenfold cross-validation using the minimal criteria. The patients’ risk scores were computed using the normalized expression levels of each gene and the related regression coefficients (Liang et al., 2020). Based on the median value of the risk score, the patients were divided into high-risk and low-risk groups. PCA was performed using the “prcomp” function of the “stats” R package based on the expression of genes in the signature. Furthermore, t-distributed stochastic neighbor embedding (t-SNE) was used to investigate the distribution of distinct groups using the “Rtsne” R package. The appropriate cutoff expression value for each gene’s survival analysis was found using the “surv cutpoint” function of the “survminer” R package. To assess the prediction potential of the gene signature, time-dependent ROC curve studies were performed using the “survivalROC” R package (Heagerty et al., 2000).

### Clinical correlation of the cuproptosis-related prognostic signature

The associations between the risk score and the clinical characteristics, tumor stage, and mutations were investigated using the chi-square test. Univariate and multivariate analyses were carried out on the training and testing sets to assess whether the predictive value of the risk score was independent of other available clinical pathology parameters.

### Functional enrichment analysis

Based on the DEGs (|log2FC|>1, FDR<0.05) between the high-risk and low-risk groups, the “clusterProfiler” R package was used to perform GO and KEGG enrichment analysis and GSEA studies. The BH approach was used to adjust the *p* values.

### Immune characteristics analysis

Correlations between the expression of genes in the signature and immune cells were evaluated by the TIMER2.0 database (http://timer.cistrome.org/). Single-sample gene set enrichment analysis (ssGSEA) in the “gsva” R package was used to determine the infiltration score of 16 immune cells and the activity of 13 immune-related pathways (Rooney et al., 2015). Immune cell infiltration and stromal cell infiltration were quantified with the “ESTIMATE” R package. The proportion of 22 immune cells was estimated by the “CIBERSORT” algorithm.

### Drug sensitivity

To explore the drug sensitivity of the two groups of patients, the “Oncopredict” software package was used to calculate the half-maximal inhibitory concentration (IC50) values of LGG after multidrug treatment (Maeser et al., 2021). The probability of an immunotherapy response was calculated using the TIDE method (Jiang et al., 2018).

### Statistical analysis

The gene expression of tumor tissues and adjacent nontumorous tissues was compared using Student’s t test. The chi-squared test was used to compare proportional differences. The ssGSEA scores of immune cells or pathways were compared between the high-risk and low-risk groups using the Mann–Whitney test with *p* values corrected using the BH technique. The log-rank test was used to compare the OS of various groups using Kaplan-Meier analysis. To identify independent determinants of OS, researchers used univariate and multivariate Cox regression analyses. R software (Version 3.5.3) or SPSS was used for all statistical analyses (Version 23.0). A *p* value of less than 0.05 was regarded as statistically significant unless otherwise stated, and all *p* values were two-tailed.

## Results

**FIGURE 1:**
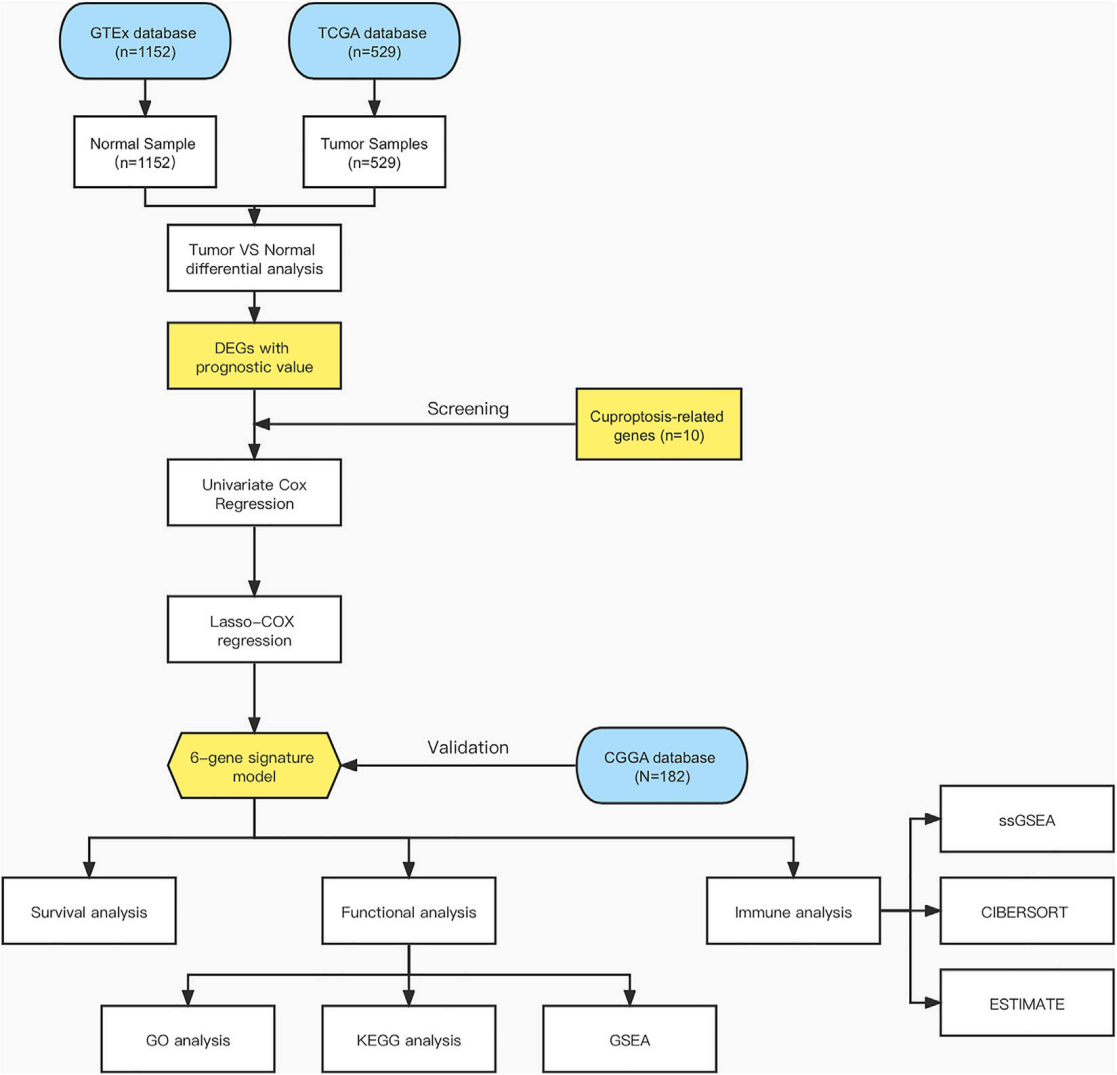
Flow chart of data collection and analysis.

Figure 1 shows a flow chart depicting the steps of this investigation. A total of 529 LGG patients from the TCGA-LGG cohort and 182 LGG patients from the CGGA cohort were finally enrolled. The detailed clinical characteristics of these patients are summarized in Table 1 and Table 2.

**TABLE 1:** Clinical characteristics of the LGG patients from the TCGA cohort.

Characteristic	High	Low	p
n	262	262	
Gender, n (%)			0.926
Female	105 (22.6%)	103 (22.2%)	
Male	127 (27.4%)	129 (27.8%)	
Grade, n (%)			0.020
G2	98 (21.1%)	124 (26.7%)	
G3	134 (28.9%)	108 (23.3%)	
IDH status, n (%)			<0.001
Mutant	181 (34.7%)	243 (46.6%)	
WT	79 (15.2%)	18 (3.5%)	
1p/19q codeletion, n (%)			<0.001
codel	12 (2.3%)	156 (29.8%)	
non-codel	250 (47.7%)	106 (20.2%)	
Age, meidan (IQR)	40 (32,24)	41.5 (33,53)	0.766

**TABLE 2:** Clinical characteristics of the LGG patients from the CGGA cohort.

Characteristic	High	Low	p
n	46	126	
Gender, n (%)			0.764
Female	19 (11%)	47 (27.3%)	
Male	27 (15.7%)	79 (45.9%)	
Grade, n (%)			<0.001
G2	16 (9.3%)	82 (47.7%)	
G3	30 (17.4%)	44 (25.6%)	
IDH status, n (%)			<0.001
Mutant	25 (14.6%)	102 (59.6%)	
WT	21 (12.3%)	23 (13.5%)	
1p/19q codeletion, n (%)			<0.001
codel	0 (0%)	55 (32.4%)	
non-codel	46 (27.1%)	69 (40.6%)	
Age, mean ± SD	43.54 ± 13.54	39.32 ± 9.49	0.056

### Identification of prognostic cuproptosis-related DEGs in the TCGA cohort

**FIGURE 2:**
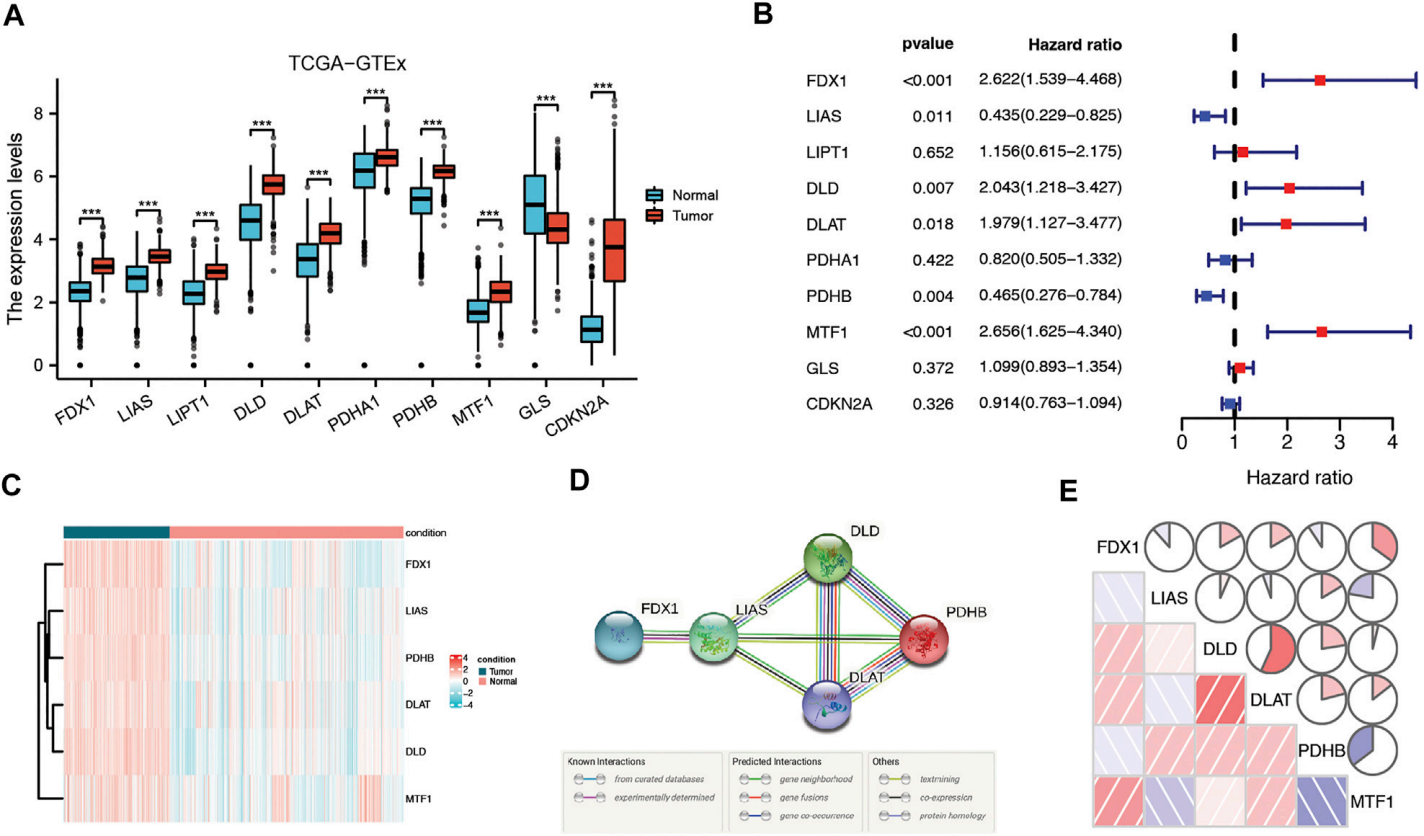
Identification of candidate cuproptosis-related genes in the TCGA cohort. **(A)** Cuproptosis-related genes were differentially expressed between glioma tissue and normal tissue. **(B)** Forest plots showing the results of the univariate Cox regression analysis of DEGs related to OS. **(C)** Heatmap showing the expression of cuproptosis-related prognostic genes in tumor and normal tissues. **(D)** The PPI network downloaded from the STRING database indicated the interactions among candidate genes. **(E)** The correlation network of candidate genes. The correlation coefficients are represented by different colors. Adjusted p values are shown as follows: ns, not significant; *, p < 0.05; **, p < 0.01; ***, p < 0.001.

According to previous research, ten genes were found to be related to cuproptosis: FDX1, LIAS, LIPT1, DLD, DLAT, PDHA1, PDHB, MTF1, GLS, and CDKN2A(8). Based on the TCGA and GTEx databases, all 10 genes were differentially expressed between tumor tissues and normal tissue, and of these, six genes (FDX1, LIAS, DLD, DLAT, PDHB, and MTF1) were correlated with LGG patient prognosis according to Cox regression analysis (Figures 2A,B) Therefore, six prognostic cuproptosis-related DEGs were identified. The heatmap showed that these genes were upregulated in tumor tissue (Figure 2C) The interaction network involving these genes indicated that DLD, DLAT, and PDHB were the hub genes (Figure 2D). The correlation between these genes is presented in Figure 2E.

### Construction of a cuproptosis-related genes signature

Using the LASSO approach, we aimed to identify a prognostic gene set for LGG. The abovementioned genes were used to develop a prognostic signature, and a regression model with a minimal lambda was used to generate a gene signature that could be quantified in each patient (Supplementary Figure S1A,B). With this, we created a scoring system that we named the CRG risk score to calculate the CRG pattern for a specific patient. The following formula was used to determine the risk score: e(0.38*expression level of FDX1-0.39*expression level of LIAS+0.41*expression level of DLD+0.08*expression level of DLAT-0.4*expression level of PDHB+0.45*expression level of MTF1).

### Prognostic value of the cuproptosis-related genes signature in the the cancer genome atlas cohort

**FIGURE 3:**
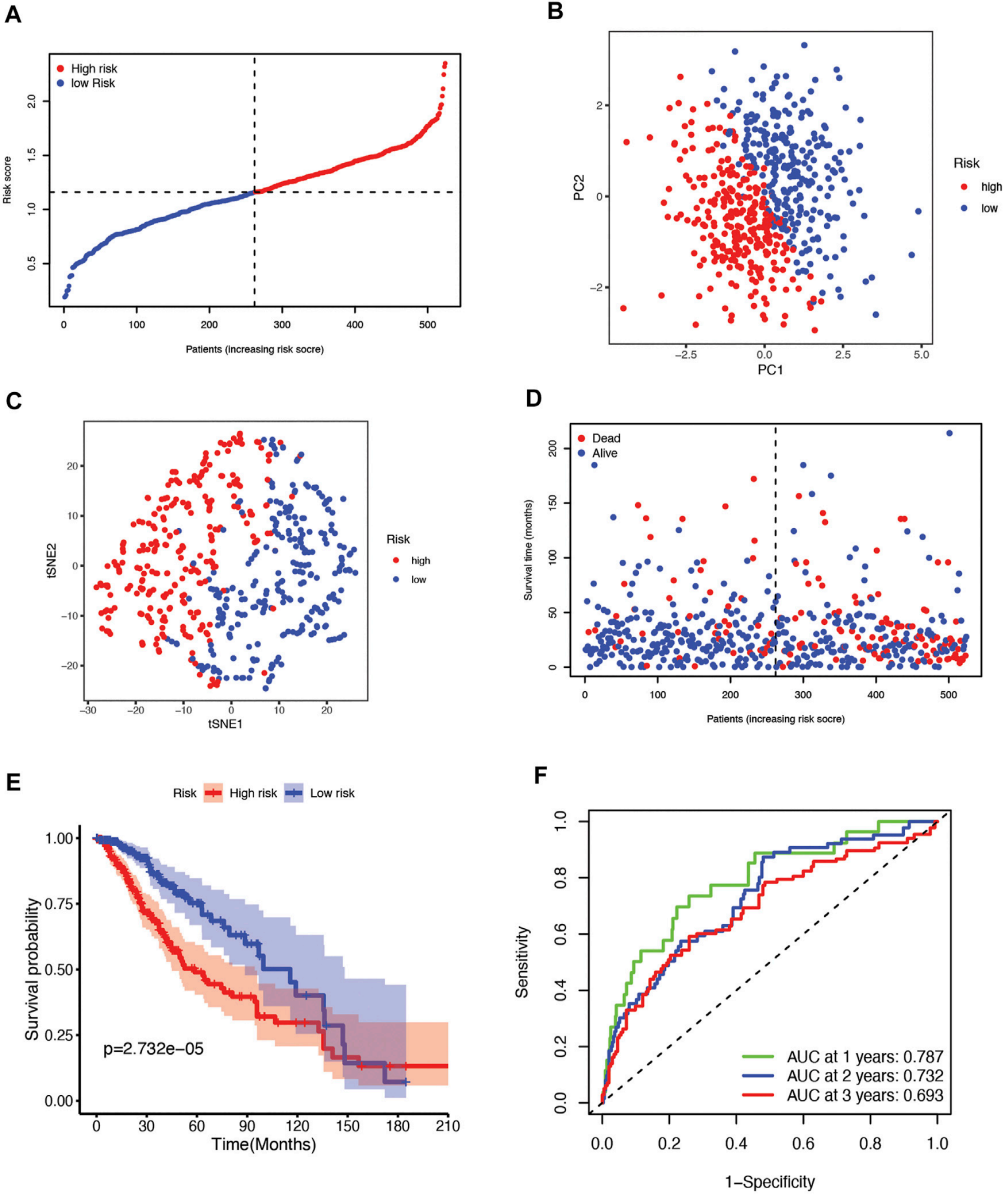
**(A)** The distribution and median value of the risk scores in the TCGA cohort. **(B)** PCA plot of the TCGA cohort. **(C)** t-SNE analysis of the TCGA cohort. **(D)** The distribution of OS status, OS, and risk score in TCGA. **(E)** Kaplan‒Meier curves for the OS of patients in the high-risk group and low-risk group in the TCGA cohort. **(F)** The AUC values of time-dependent ROC curves verified the prognostic performance of the risk score in the TCGA cohort.

According to the median cutoff value, which is 1.160294, the patients were divided into two groups: high-risk (n = 262) and low-risk (n = 262) (Figure 3A). The patients in distinct risk groups were clustered separately according to PCA and t-SNE analyses (Figures 3B,C). Patients with higher risk scores were highly likely to have higher mortality risks and shorter survival times (Figure 3D). The Kaplan–Meier curve consistently showed that patients in the high-risk group had a considerably shorter OS than those in the low-risk group (Figure 3E). Time-dependent ROC curves were used to assess the risk score’s predictive ability for OS, and the area under the curve (AUC) reached 0.787 at 1 year, 0.732 at 2 years, 0.693 at 3 years (Figure 3F), 0.684 at 4 years, and 0.693 at 5 years (Supplementary Figure S2A).

### Prognostic value of the cuproptosis-related genes signature in the chinese glioma genome atlas cohort

**FIGURE 4:**
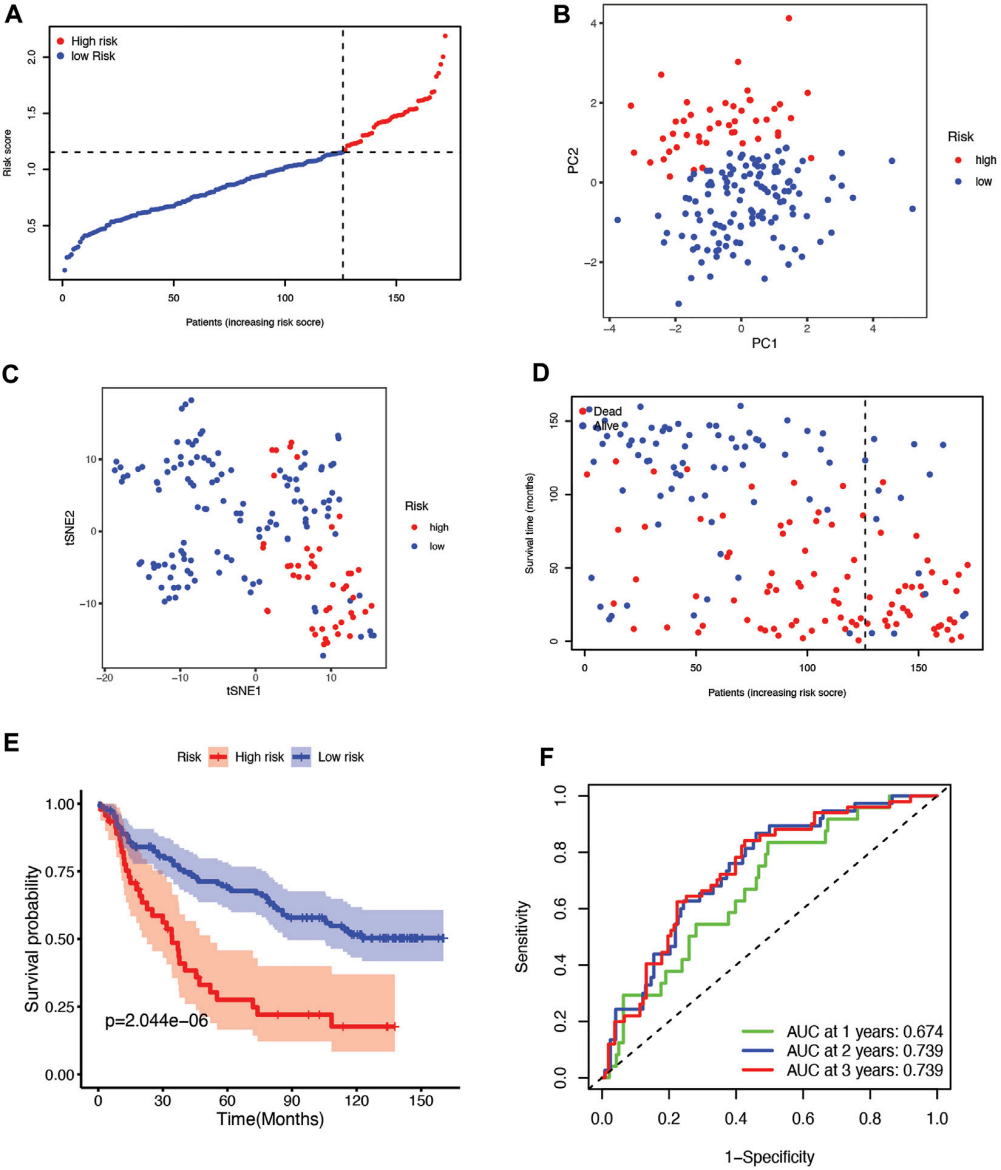
**(A)** The distribution and median value of the risk scores in the CGGA cohort. **(B)** PCA plot of the CGGA cohort. **(C)** t-SNE analysis of the CGGA cohort. **(D)** The distribution of OS status, OS, and risk score in the CGGA. **(E)** Kaplan‒Meier curves for the OS of patients in the high-risk group and low-risk group in the CGGA cohort. **(F)** The AUCs of time-dependent ROC curves verified the prognostic performance of the risk score in the CGGA cohort.

To validate the risk signature developed based on the TCGA cohort, patients from the CGGA cohort were likewise divided into high-risk and low-risk groups according to the median value obtained with the formula based on the TCGA cohort (Figure 4A). PCA and t-SNE analyses indicated that patients in the two subgroups were clustered separately, similar to the results for the TCGA cohort (Figure 4B,C). Patients in the high-risk group were also more likely to die early (Figure 4D) and had a shorter survival time (Figure 4E) than those in the low-risk group. Furthermore, the AUCs of the six-gene signature were 0.674, 0.739, 0.739, 0.782, and 0.797 from 1 year to 5 years (Figure 4F, Supplementary Figure S2B).

### Assessment of the independence of the cuproptosis-related genes signature

**FIGURE 5:**
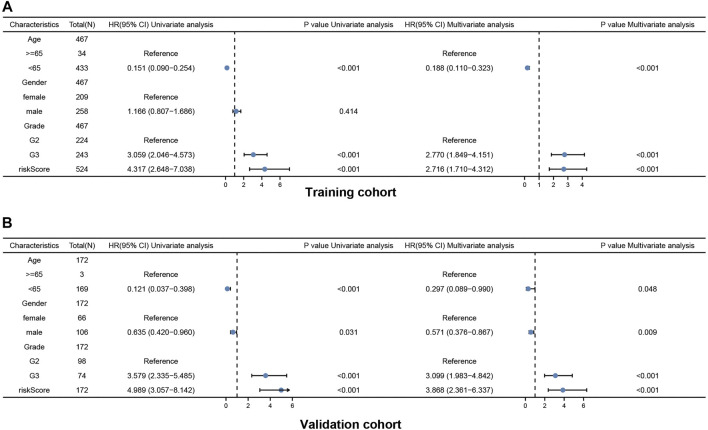
Results of the univariate and multivariate Cox regression analyses regarding OS in the TCGA cohort **(A)** and the CGGA validation cohort **(B)**.

**FIGURE 6:**
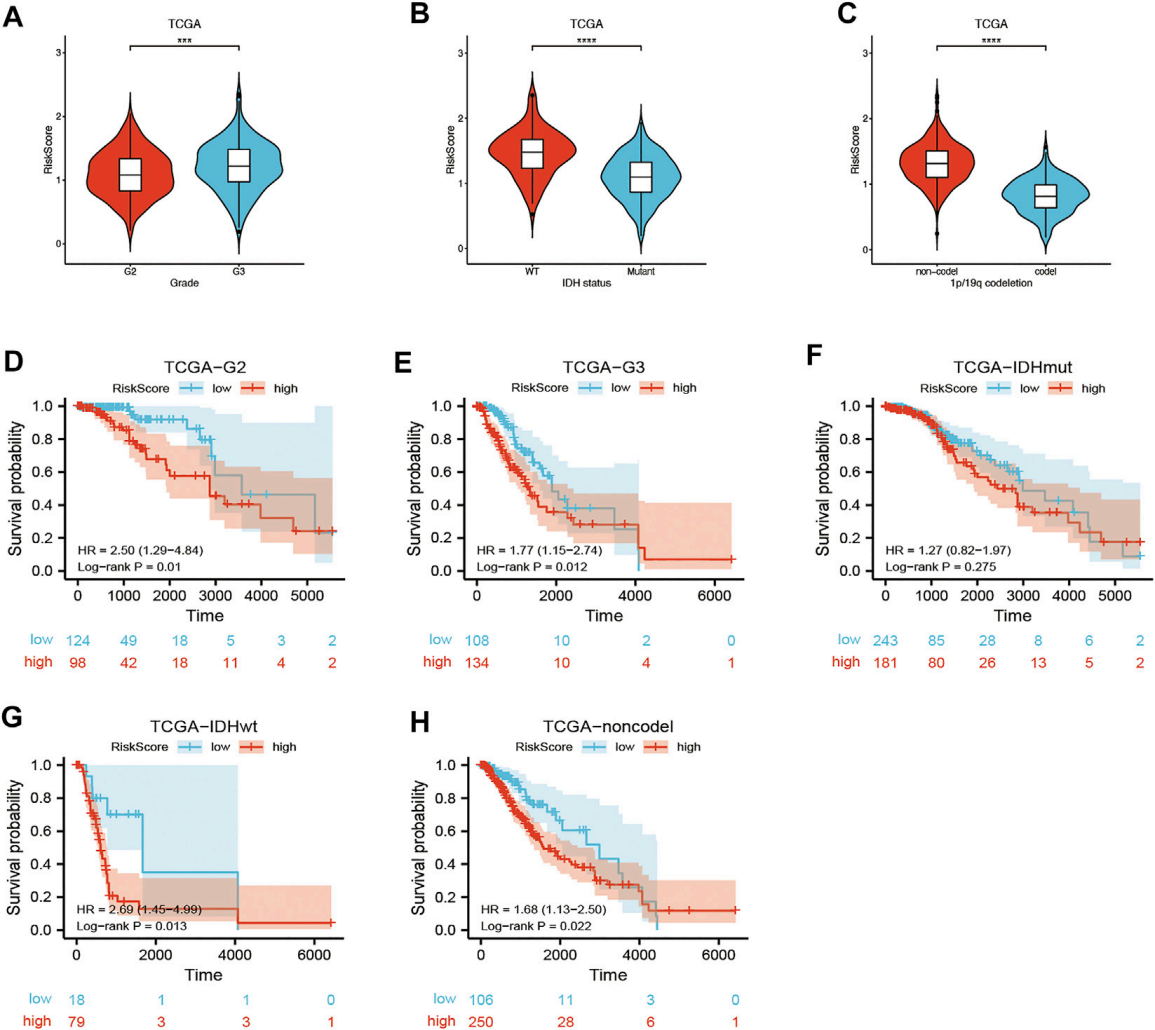
Kaplan–Meier curve of stratified analyses of the CRG signature for associations with clinical characteristics in the TCGA cohort. **(A)** Risk score between grade 2 and grade 3 stage patients. **(B)** Risk score between IDH mutation and IDH wild-type patients. **(C)** Risk score between 1q/19p non-codeletion and 1q/19p codeletion patients. **(D)** OS curve in grade 2 patients. **(E)** OS curve in grade 3 patients. **(F)** OS curve in IDH mutation patients. **(G)** OS curve in IDH wild-type patients. **(H)** OS curve in 1q/19p non-codeletion patients. Adjusted p values are shown as follows: ns, not significant; *, p < 0.05; **, p < 0.01; ***, p < 0.001.

**FIGURE 7:**
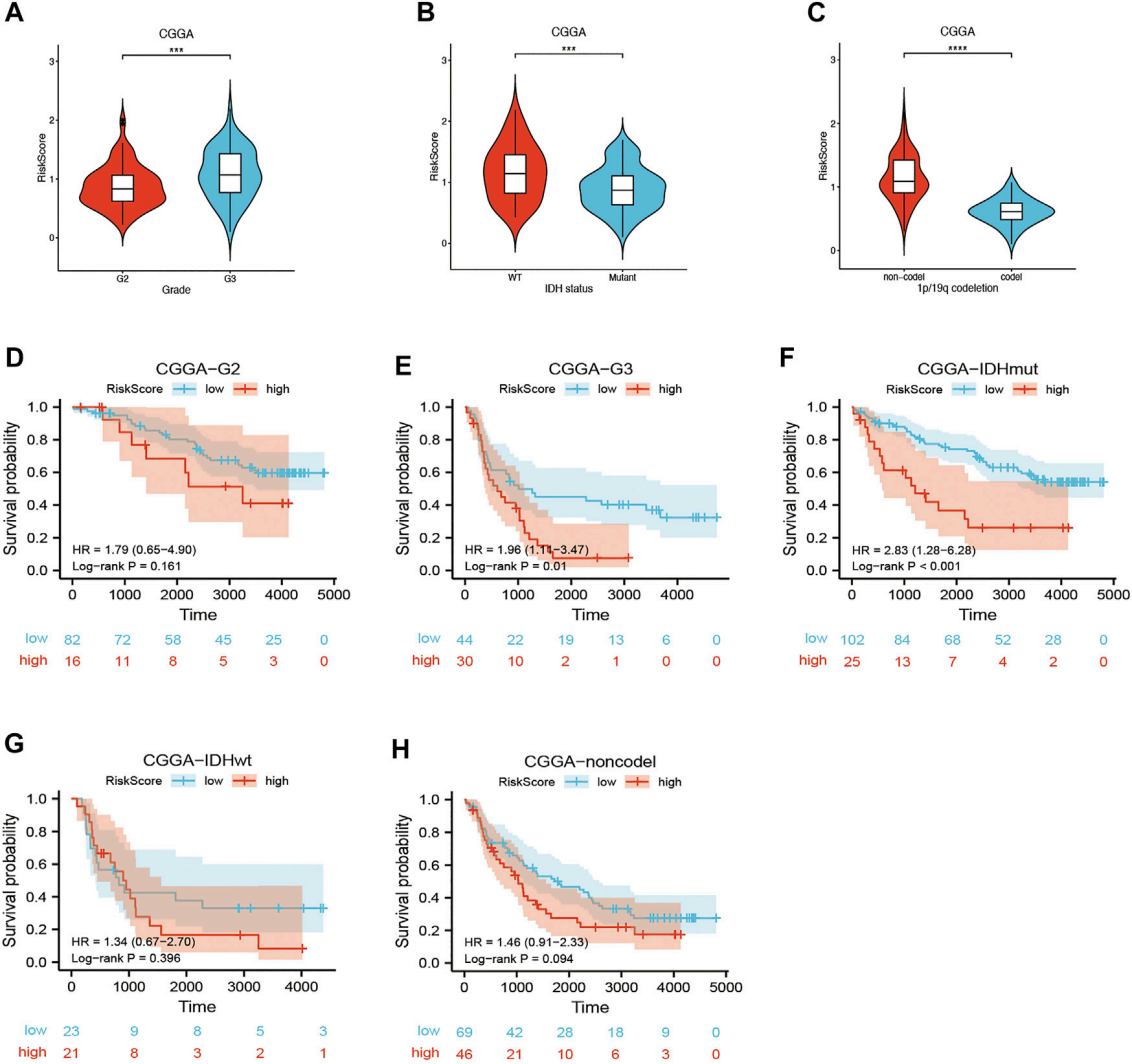
Kaplan–Meier curve of stratified analyses of the CRG signature for associations with clinical characteristics in the CGGA cohort. **(A)** Risk score between grade 2 and grade 3 stage patients. **(B)** Risk score between IDH mutation and IDH wild-type patients. **(C)** Risk score between 1q/19p non-codeletion and 1q/19p codeletion patients. **(D)** OS curve in grade 2 patients. **(E)** OS curve in grade 3 patients. **(F)** OS curve in IDH mutation patients. **(G)** OS curve in IDH wild-type patients. **(H)** OS curve in 1q/19p non-codeletion patients. Adjusted p values are shown as follows: ns, not significant; *, p < 0.05; **, p < 0.01; ***, p < 0.001.

Univariate and multivariate Cox regression analyses of the available factors were performed to evaluate whether the risk score was an independent prognostic predictor of OS. In both the TCGA and CGGA cohorts, the risk score was substantially correlated with OS in the univariate Cox regression models (TCGA cohort: HR = 4.317, 95% CI = 2.648–7.038, *p* < 0.001; CGGA cohort: HR = 4.989, 95% CI = 3.057–8.142, *p* < 0.001) (Figures 5A,B). In the multivariate Cox regression analysis, the risk score remained an independent predictor of OS after controlling for additional confounding variables (TCGA cohort: HR = 2.716, 95% CI = 1.710–4.312, *p* < 0.001; CGGA cohort: HR = 3.868, 95% CI = 2.361–6.337, *p* < 0.001; Figures 5A,B). Meanwhile, stratified analyses were performed to investigate the correlation between clinical characteristics and CRG risk scores. The results showed that patients with advanced stage, wild-type IDH status, or 1p/19q non-codeletion LGG were more likely to have a higher risk score than those who had a lower stage and mutation type LGG in both TCGA (Figures 6A–C) and CGGA cohort (Figures 7A–C). The prognostic risk signature accurately divided LGG patients with different clinical features into short-term and long-term survival groups according to the Kaplan‒Meier plot. In both the TCGA and CGGA cohorts, the G2, G3, IDH mutation, IDH wild type, and 1p/19q non-codeletion groups, who had lower risk scores, survived longer than those who had higher risk scores (Figures 6D–H, Figures 7D–H). In addition, consistent results were obtained when risk of patient was determined according to the clinical subgroups median risk score (Supplementary Figures S3, S4).

### Functional annotation of the cuproptosis-related genes signature in the the cancer genome atlas and chinese glioma genome atlas cohorts

**FIGURE 8:**
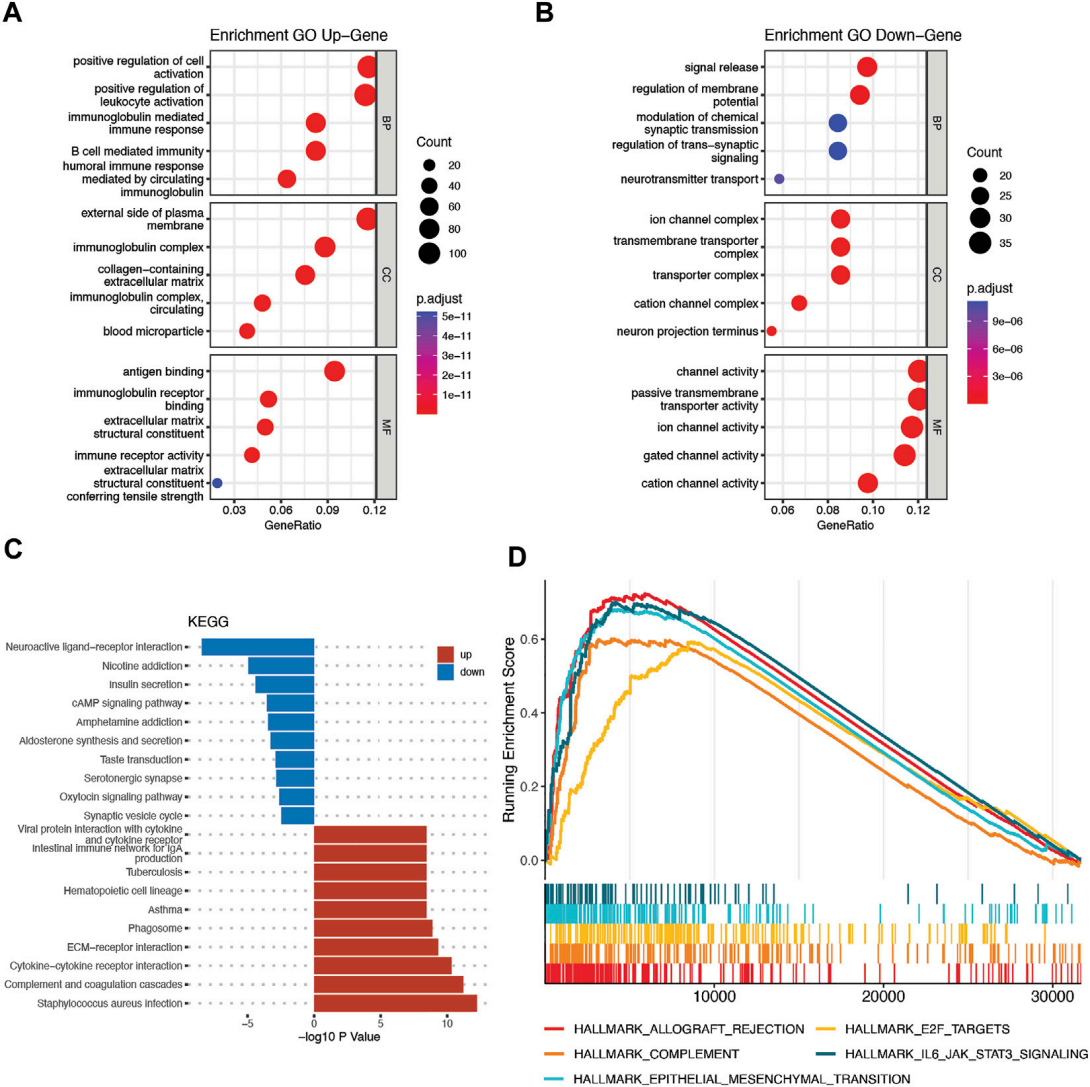
Functional analysis of DEGs between the high- and low-risk groups in the TCGA cohort. **(A and B)** Bubble graph for GO analysis. **(C)** Bar plot for KEGG pathways. **(D)** GSEA.

**FIGURE 9:**
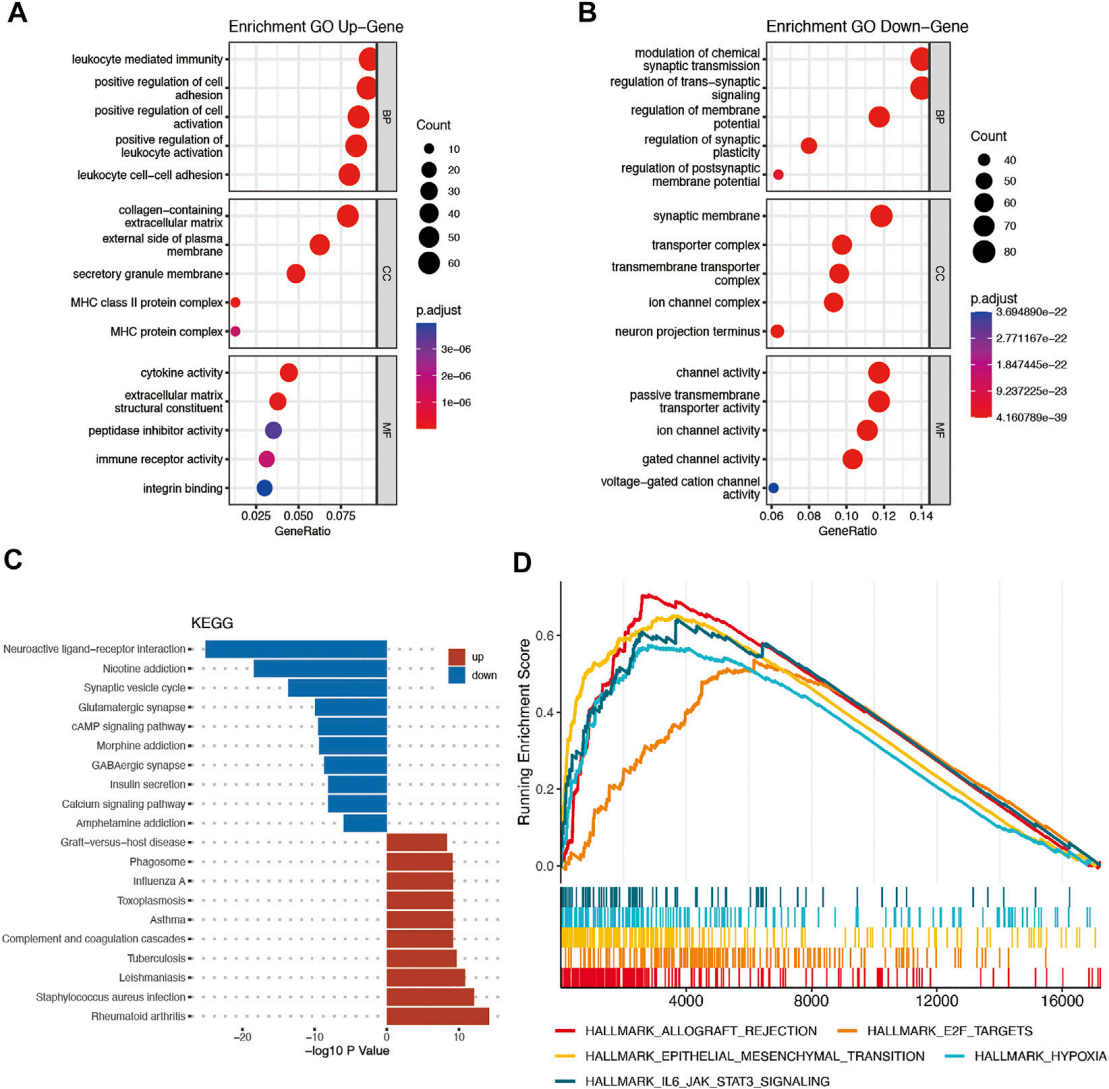
Functional analysis of DEGs between the high- and low-risk groups in the CGGA cohort. **(A and B)** Bubble graph for GO analysis. **(C)** Bar plot for KEGG pathways. **(D)** GSEA.

The DEGs between the high-risk and low-risk groups were utilized to conduct GO enrichment and KEGG pathway analyses to reveal the biological activities and pathways related to the risk score. In both the TCGA and CGGA cohorts, DEGs were enriched in molecular functions and cellular component terms relevant to substance transportation, such as ion channel activity and ion channel complex. Interestingly, the TCGA cohort DEGs were clearly enriched in immune-related biological processes, such as immunoglobulin-mediated immune response, B-cell-mediated immunity, and humoral immune response mediated by circulating immunoglobulin (Figures 8A,B). Analysis of the CGGA cohort corroborated these findings. The DEGs were enriched in leukocyte-mediated immunity and positive regulation of leukocyte activation (Figures 9A,B). The neuroactive ligand‒receptor interaction and cAMP signaling pathways were also shown to be enriched in DEGs from both the TCGA and CGGA cohorts, according to KEGG pathway analyses (Figures 8C, Figure 9C). GSEA showed that the IL6-JAK-STAT3 signaling pathway was enriched in DEGs from both the TCGA and CGGA cohorts (Figures 8D, Figure 9D).

### Comparison of immune characteristics between subgroups

**FIGURE 10:**
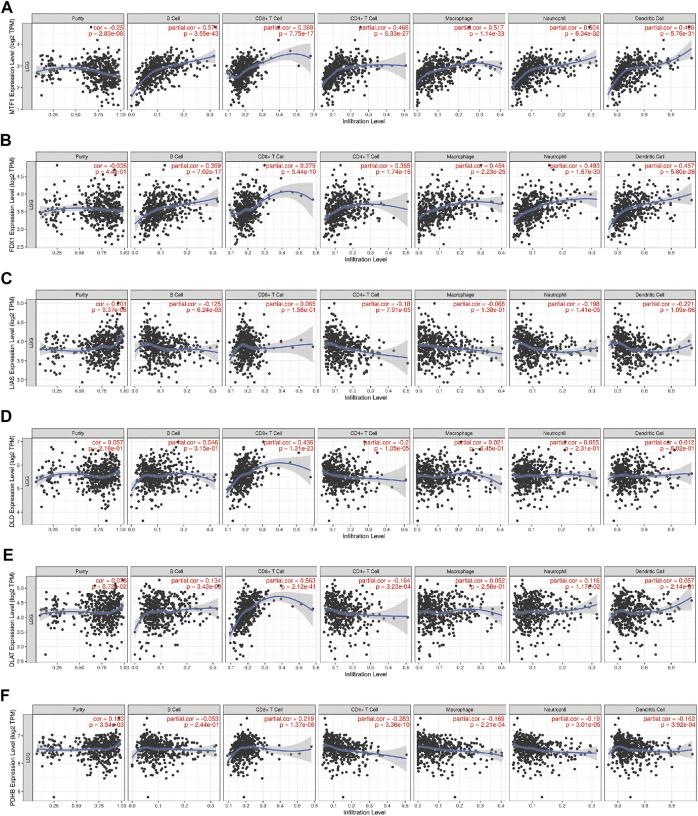
The correlations between the expression of 6 CRGs and the levels of immune cells from the TIMER database. **(A)** MTF1 and immune cells; **(B)** FDX1 and immune cells; **(C)** LIAS and immune cells; **(D)** DLD and immune cells; **(E)** DLAT and immune cells; **(F)** PDHB and immune cells.

**FIGURE 11:**
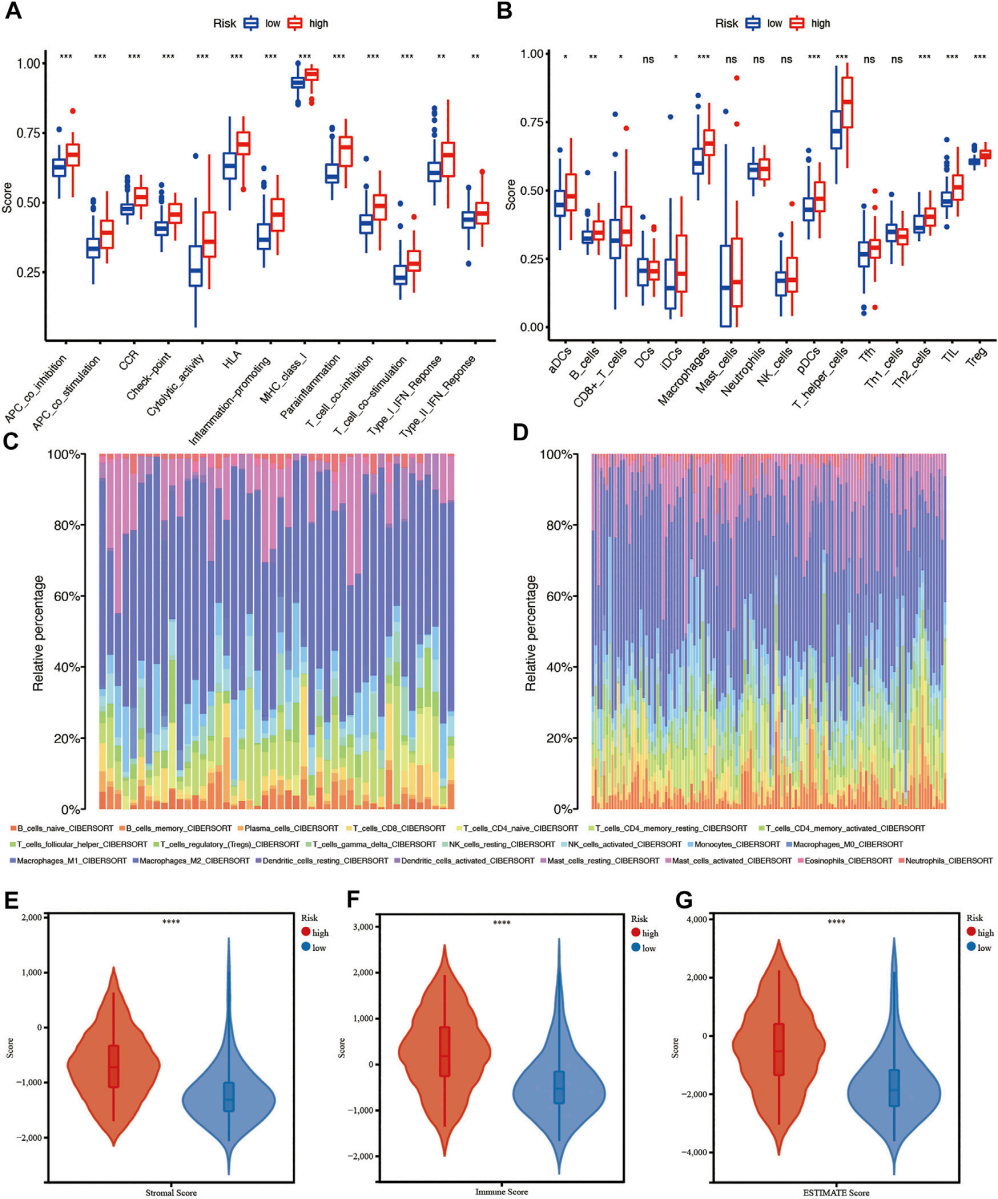
Immune characteristics analysis in the CGGA cohort. **(A and B)** ssGSEA scores of 13 immune-related functions and scores of 16 immune cells between the high- and low-risk groups. **(C and D)** Immune cell infiltration in the high- and low-risk groups using CIBERSORT. **(E–G)** Immune score, stromal score, and combined score estimated using ESTIMATE. Adjusted *p* values are shown as follows: ns, not significant; *, *p* < 0.05; **, *p* < 0.01; ***, *p* < 0.001.

First, to investigate the relationship between a single CRG and immune infiltration, 6 CRGs were independently imported into the TIMER database (Figure 10). The results showed that MTF1 and FDX1 were closely associated with immune infiltration. The expression of MTF1 and FDX1 was positively related to B cells, CD8^+^ T cells, CD4^+^ T cells, macrophages, neutrophils, and dendritic cells (Figures 10A,B). However, PDHB was negatively related to the cells mentioned above (Figure 10F). Next, we used ssGSEA, CIBERSORT, and ESTIMATE to quantify the enrichment scores of various immune cell subpopulations, associated functions, and pathways to further investigate the relationship between the risk score and immunological state. Surprisingly, all the immune-related functions and pathways differed between the low-risk and high-risk groups in the CGGA cohorts (Figure 11A). In the CGGA cohort, B cells, CD8^+^ T cells, immature DCs, macrophages, plasmacytoid DCs, T helper cells, Th2 cells, tumor-infiltrating lymphocytes (TILs), and Tregs were differentially abundant between the high-risk group and the low-risk group (Figures 11B–D). Based on immune scores, macrophages and T helper cells were most significantly differentially abundant between the high-risk group and low-risk groups, which was consistent with the outcomes from GO and KEGG analyses. Furthermore, ESTIMATE analysis showed that the higher the risk score was, the higher the stromal, immune, and estimate scores were (Figures 11E–G).

### Comparison of anticancer drug sensitivity between patients in different subgroups

**FIGURE 12:**
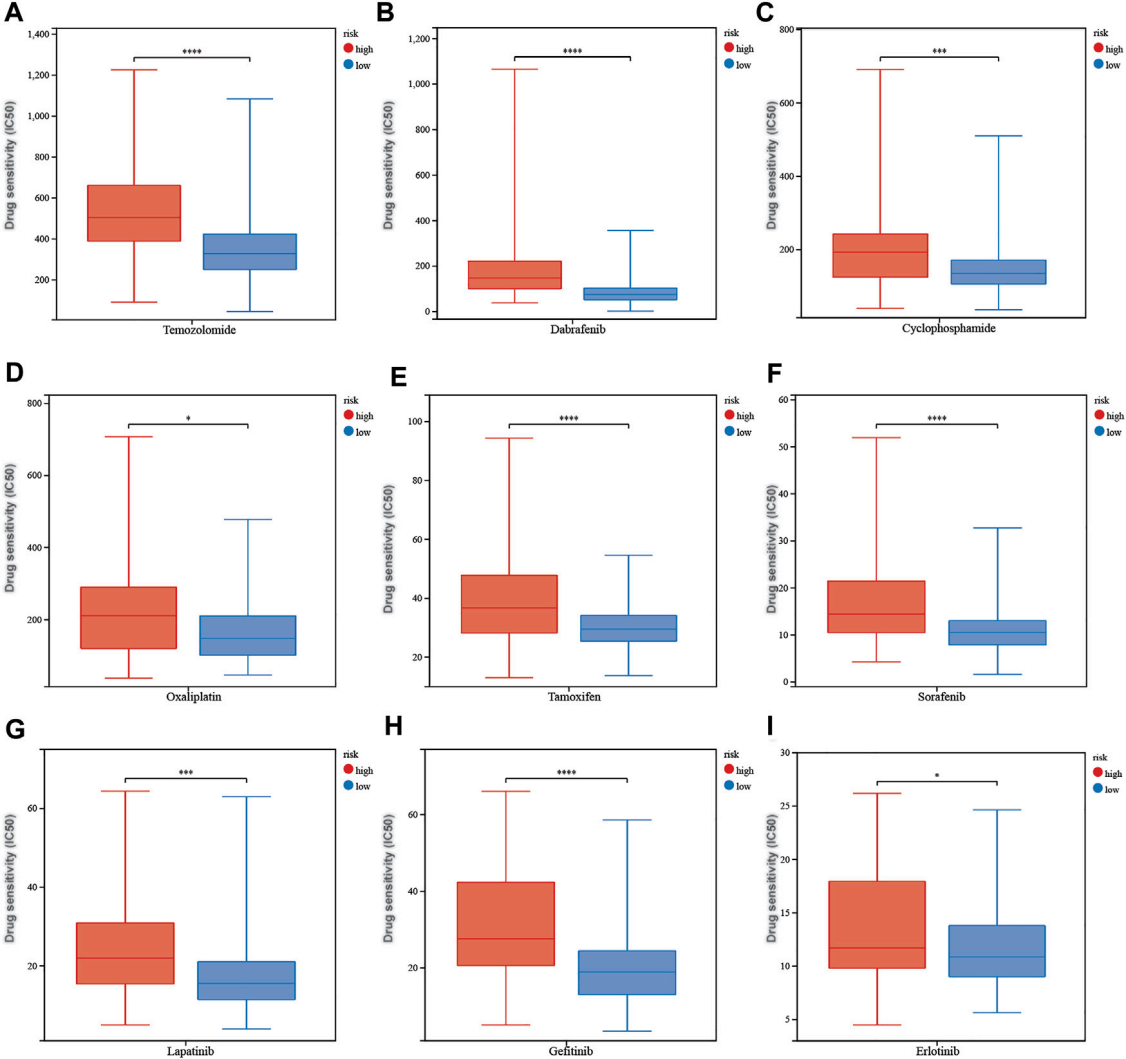
The correlation between different risk groups and drug sensitivity in LGG patients. **(A)** temozolomide, **(B)** dabrafenib, **(C)** cyclophosphamide, **(D)** oxaliplatin, **(E)** tamoxifen, **(F)** sorafenib, **(G)** lapatinib, **(H)** gefitinib, and **(I)** erlotinib. Adjusted p values are shown as follows: ns, not significant; *, p < 0.05; **, p < 0.01; ***, p < 0.001.

**FIGURE 13:**
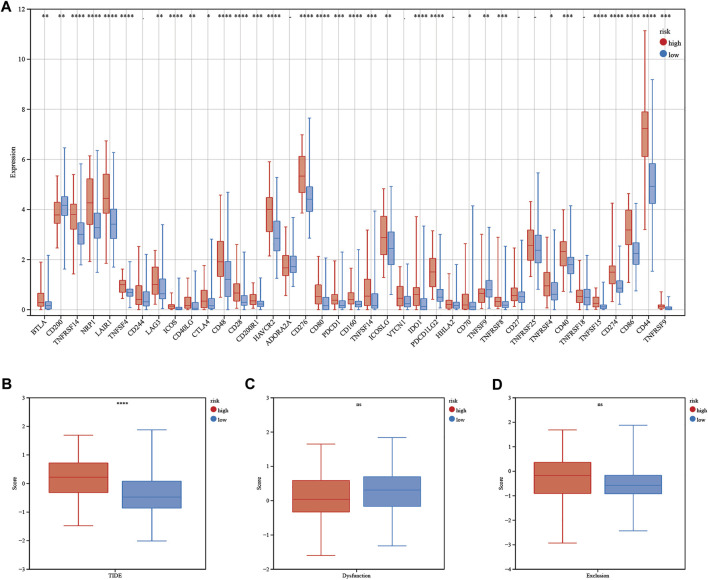
Immunotherapy response of LGG patients. **(A)** Differences in immune checkpoint gene expression between patients in the high- and low-risk groups. **(B–D)** TIDE, dysfunction and exclusion scores between patients in the high- and low-risk groups. Adjusted p values are shown as follows: ns, not significant; *, p < 0.05; **, p < 0.01; ***, p < 0.001.

The calculated half-maximal inhibitory concentration (IC50) differed significantly across the two risk groups. Interestingly, the IC50 values of temozolomide, dabrafenib, cyclophosphamide, oxaliplatin, tamoxifen, sorafenib, lapatinib, gefitinib, and erlotinib were lower in patients with low CRG risk scores, suggesting that the CRGs are correlated with drug resistance (Figure 12). Immune checkpoint-related gene expression was higher in the high-risk group than in the low-risk group, demonstrating that the risk score matched the status of tumor-induced immunosuppression (Figure 13A). The TIDE algorithm was used to predict the clinical outcome of immune checkpoint inhibitors. TIDE scores were substantially different between the high-risk and low-risk groups (Figure 13B). According to the TIDE analysis, low-risk patients had a higher exclusion score and a higher dysfunction score than high-risk patients (Figures 13C,D).

## Discussion

Glioma is a malignant brain tumor that has a significant impact on human health. WHO grade II and grade III gliomas are considered LGGs. Untreated LGG spontaneously progresses into higher-grade glioma on average 4–5 years after diagnosis. However, glioma also tends to relapse despite rigorous treatment, such as surgery, radiotherapy, chemotherapy, and targeted therapy (Chang et al., 2016). These findings indicate that there is an urgent need to predict the prognosis of LGG patients. In recent years, programmed cell death, apoptosis, pyroptosis, and ferroptosis have been regarded as potential options for treating cancer. Therefore, cuproptosis, as a novel form of regulated cell death triggered by excessive copper, is of great value to study (Tang et al., 2022). Recently, cuproptosis has attracted much attention, as it could be a factor that has a huge impact on cancer development and progression. Many studies have applied CRGs to predict outcomes and guide treatments for cancer patients, for example, triple-negative breast cancer, hepatocellular carcinoma, and bladder cancer patients. As shown in previous studies, CRGs are closely related to the prognosis of breast cancer patients (Sha et al., 2022). In addition, the cuproptosis-related risk score could divide liver cancer patients into two groups with differences in mutation status and responded to target therapy in different ways (Zhang et al., 2022). Furthermore, cuproptosis patterns were able to predict immunotherapy responses in bladder cancer patients (Song et al., 2022). A previous study proved that cuproptosis plays an essential role in glioma growth and progression (Buccarelli et al., 2021). Moreover, Chen et alestablished a cuproptosis activity score (CuAS) based on bulk tumor and single-cell transcriptome data, suggesting that cuproptosis mediates glioma aggressiveness and neoplasm-immune interactions (Chen et al., 2022). In addition, Yan et alreported that cuproptosis-related lncRNAs can guide LGG treatment, as they could divide LGG patients into different groups according to the response to immunotherapy (Yan et al., 2022). In addition, two subclusters of glioma patients were identified based on CRG expression, which was correlated with tumor driver gene mutations and the response to chemotherapy, according to Wang et al. (Wang et al., 2022). Thus, it is critical to explore the relationship between cuproptosis-related genes and LGG prognosis to achieve a comprehensive understanding of the function of cuproptosis in LGG and thereby further improve the efficacy of prognosis and treatment options for gliomas. In the current study, we focused on the expression of 10 CRGs in LGG tumor tissues and their relationships with OS. Surprisingly, all cuproptosis-related genes were differentially expressed between tumor and normal tissues, and six of them were correlated with OS in the univariate Cox regression analysis. First, a novel prognostic signature including six cuproptosis-related genes was developed and tested in the TCGA and CGGA databases. As shown above, the prognostic signature separated patients into high-risk and low-risk groups, and this separation was validated by PCA and t-SNE analysis. The overall survival of patients in the high-risk group was significantly shorter than that of patients in the low-risk group. These findings strongly suggested that differentially expressed cuproptosis-related genes affected LGG development and progression. The prognostic signature included 6 genes (FDX1, LIAS, DLD, DLAT, PDHB, and MTF1). According to *Golub* et al., cuproptosis-related genes are either correlated with the lipoic acid (LA) pathway or involved in the formation of the pyruvate dehydrogenase (PDH) complex (Tsvetkov et al., 2022). FDX1 is a crucial factor in the synthesis of steroid hormones and heme A and Fe/S clusters (Sheftel et al., 2010). In addition, a recent study revealed that FDX1 is involved in glucose metabolism, fatty acid oxidation, and amino acid metabolism (Zhang et al., 2021). Importantly, FDX1 could be a target of cuproptosis, as it is directly regulated by elesclomol (Tsvetkov et al., 2019). The final step in the production of lipoic acid, an antioxidant, is catalyzed by LIAS. According to previous research, overexpression of LIAS in a mouse model enhanced antioxidant defense and proved effective in treating diabetic nephropathy, nonalcoholic fatty liver disease, pulmonary fibrosis, and atherosclerosis (Tian et al., 2020; Zhao et al., 2020; Xu et al., 2021; Zhao et al., 2021). The pyruvate dehydrogenase complex (PDC) is essential for glucose metabolism, as it connects glycolysis to the TCA cycle (Echeverri Ruiz et al., 2021). The E1, E2, and E3 subunits of the PDC are encoded by PDHB, DLAT, and DLD, respectively (Goguet-Rubio et al., 2016). DLD, a redox enzyme, plays an important role in glucose metabolism and ATP production. Under certain circumstances, it can either stimulate or inhibit ROS and reactive nitrogen species production. Thus, DLD inhibitors could be used to protect against oxidative damage (Yang et al., 2019). Moreover, DLD inhibition increases intracellular ROS generation and lowers mitochondrial membrane potential, resulting in autophagic cell death (Dayan et al., 2019). DLAT is found to regulate cell proliferation and carbohydrate metabolism in gastric cancer cells and B-cell chronic lymphocytic leukemia cells (Goh et al., 2015; Mayer et al., 2018). Overexpression of PDHB switches cell metabolism from glycolysis to the Krebs cycle in gastric cancer and epithelial ovarian carcinoma (Cai et al., 2010; Li et al., 2020). It has been reported that PDHB exerts an anticancer effect by counteracting oncogenic noncoding RNAs, such as miR-370, miR-363-3p, and miR-146b-5p, in melanoma, glioma, and colorectal cancer, respectively (Wei and Ma, 2017; Zhu et al., 2017; Xu et al., 2018). MTF1 is a transcription factor that responds to metal excess or deficiency and shields cells from oxidative and hypoxic damage (Tavera-Montañez et al., 2019). MTF1 inhibits matrix collagen deposition and stimulates angiogenesis to promote tumor growth (Haroon et al., 2004). According to a previous study, knockout of MTF1 inhibits ovarian cancer cell proliferation, migration, and invasion by suppressing epithelial-to-mesenchymal transition (Ji et al., 2018).

Functional analyses, such as GO and KEGG analyses, were performed to explain the underlying mechanism of prognosis between the high-risk and low-risk groups. As shown in the GO analysis, cellular components and molecular functions related to substance transportation were downregulated in the high-risk groups. We assumed that copper was more difficult to transport into cells in high-risk group samples, thus inhibiting cuproptosis. In addition, many immune-related biological processes and pathways were enriched. It is reasonable to assume that cuproptosis may have a close connection with tumor immunity. According to TIMER analysis, each of six CRGs are related to immune cell infiltration, especially MTF1 and FDX1. In addition, all six CRGs were associated with CD8^+^ T cells. Thus, to evaluate the immune activities between the high-risk and low-risk groups, the infiltration scores of 16 immune cells and the activity of 13 immune-related pathways were analyzed. Surprisingly, all of the immune-related pathways and half of the immune cells were significantly different between the high-risk group and the low-risk group. It is conceivable to conclude that cuproptosis has a strong relationship with tumor immunity. As shown above, the CD8^+^ T-cell score was higher in the high-risk group than in the low-risk group. According to previous research, CCL4 is produced by naive CD8^+^ T lymphocytes, prompting microglia to produce a key LGG growth factor (CCL5), which is necessary for LGG stem cell survival and stimulating LGG growth (Guo et al., 2020). In the high-risk group, the macrophage cells gained higher scores than those in the low-risk group. Tumor-associated macrophages (TAMs), which exert procancer functions, are M2 phenotype macrophages polarized from circulating monocytes and tissue-resident macrophages (Anderson et al., 2021). M2a-like and M2c-like macrophages have been shown to increase cell invasion and tumor growth in lung cancer, whereas M1-like macrophages decrease lung cancer cell proliferation, diminish angiogenesis, and induce death, which explains why macrophages are enriched in the high-risk group (Song et al., 2021). Treg cells, characterized by FOXP3 expression, alleviate autoimmune responses and suppress antitumor immune responses (Tanaka and Sakaguchi, 2017). Treg cells intensively infiltrated gliomas and stimulated cancer growth by promoting glioma stem cell function through TGF-beta secretion (Liu et al., 2021).

Drug sensitivity prediction showed that patients in the high-risk group were not as sensitive as patients in the low-risk group to anticancer drugs. However, immune checkpoint gene expression was higher in patients in the high-risk group. TIDE analysis showed that the high-risk group possessed a higher exclusion score and lower dysfunction score, indicating that patients in the high-risk group were more likely to benefit from immune checkpoint inhibitors (ICIs). Therefore, the CRG signature may guide clinical treatment for LGG patients.

This research has several limitations. First, we built and verified our CRG signature using data from public databases. More prospective data are needed to confirm the therapeutic value of this signature. Second, cuproptosis is a novel concept that has been studied recently. The ten cuproptosis-related genes used in this study constitute a small fraction of total cuproptosis-related genes. This prognostic signature could be made more efficient as more cuproptosis-related genes are identified. In addition, the relationship between the risk score and immunological activity should be investigated experimentally.

In conclusion, our research identified a new prognostic signature based on six cuproptosis-related genes that was able to predict prognosis for LGG patients with high sensitivity and specificity. In addition, our comprehensive analysis of CRGs revealed that these genes had a significant impact on immune features, clinical features, and treatment response. These findings provide new research directions for precise treatment of LGG patients.

## Data Availability

The original contributions presented in the study are included in the article/Supplementary Material, further inquiries can be directed to the corresponding author.

## Abbreviations

LGG, Lower-grade glioma; LASSO, Least absolute shrinkage and selection operator; TCGA, The cancer genome atlas; CGGA, Chinese glioma genome atlas; DEGs, Differentially expressed genes; OS, Overall survival; ROC, Receiver operating characteristic; FDR, False discovery rate; GO, Gene Ontology; KEGG, Kyoto encyclopedia of genes and genomes; ssGSEA, Single-sample gene set enrichment analysis; PCA, Principal component analysis; t-SNE, t-distributed stochastic neighbor embedding; AUC, Area under the curve; HR, Hazard ratio; CI, Confidence interval; APC, Antigen-presenting cell; aDC, Activated dendritic cell; iDC, Immature dendritic cell; pDC, Plasmacytoid dendritic cell; TIL, Tumor-Infiltrating Lymphocyte.
