# Supplementary material for: Cuproptosis-related gene signature stratifies lower-grade glioma patients and predicts immune characteristics
Source: Front Genet. 2022 Oct 25;13:1036460. doi: 10.3389/fgene.2022.1036460 (PMC9640744; doi:10.3389/fgene.2022.1036460)
Supplement: Supplementary file 1 [file DataSheet2.PDF]

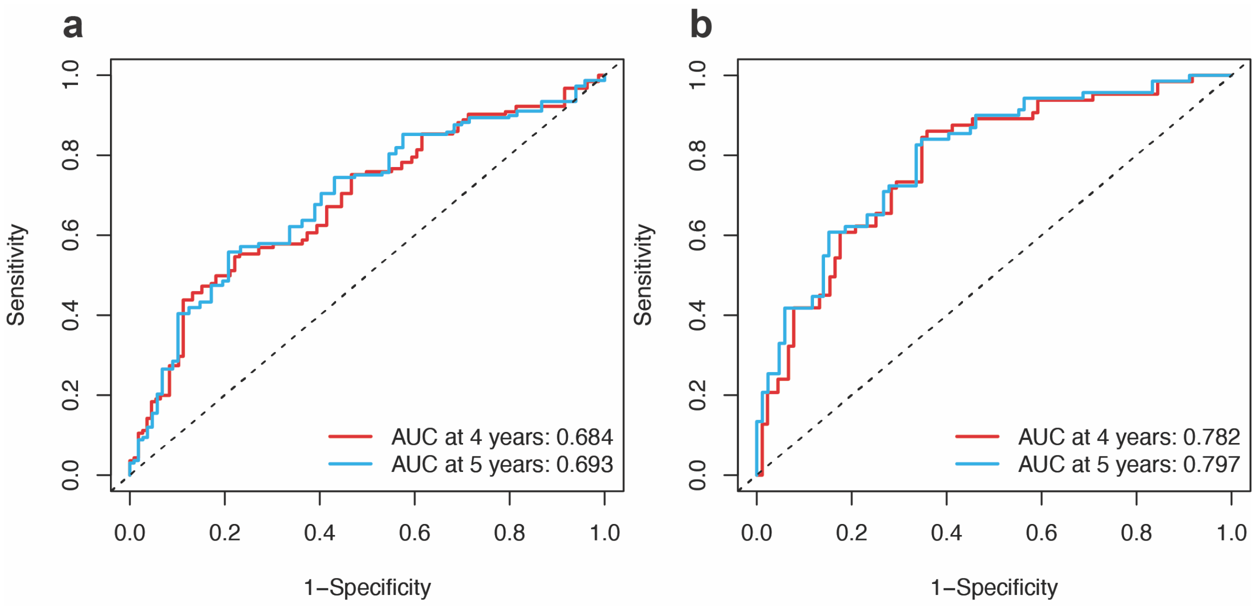

Fig. S2 AUC of time-dependent ROC curves verified the prognostic performance of the risk score in the (a) TCGA cohort and (b) CGGA cohort at 4 years and 5 years.
