# Supplementary material for: Cuproptosis-related gene signature stratifies lower-grade glioma patients and predicts immune characteristics
Source: Front Genet. 2022 Oct 25;13:1036460. doi: 10.3389/fgene.2022.1036460 (PMC9640744; doi:10.3389/fgene.2022.1036460)
Supplement: Supplementary file 3 [file Table1.DOCX]

| Characteristic | high | low | p |
| --- | --- | --- | --- |
| n | 262 | 262 |  |
| Gender, n (%) |  |  | 0.926 |
| Female | 105 (22.6%) | 103 (22.2%) |  |
| Male | 127 (27.4%) | 129 (27.8%) |  |
| Grade, n (%) |  |  | 0.020 |
| G2 | 98 (21.1%) | 124 (26.7%) |  |
| G3 | 134 (28.9%) | 108 (23.3%) |  |
| IDH status, n (%) |  |  | < 0.001 |
| Mutant | 181 (34.7%) | 243 (46.6%) |  |
| WT | 79 (15.2%) | 18 (3.5%) |  |
| 1p/19q codeletion, n (%) |  |  | < 0.001 |
| codel | 12 (2.3%) | 156 (29.8%) |  |
| non-codel | 250 (47.7%) | 106 (20.2%) |  |
| Age, meidan (IQR) | 40 (32, 54) | 41.5 (33, 53) | 0.766 |

Table 1: Clinical characteristics of the LGG patients from the TCGA cohort
