# Supplementary material for: Cuproptosis-related gene signature stratifies lower-grade glioma patients and predicts immune characteristics
Source: Front Genet. 2022 Oct 25;13:1036460. doi: 10.3389/fgene.2022.1036460 (PMC9640744; doi:10.3389/fgene.2022.1036460)
Supplement: Supplementary file 4 [file DataSheet3.PDF]

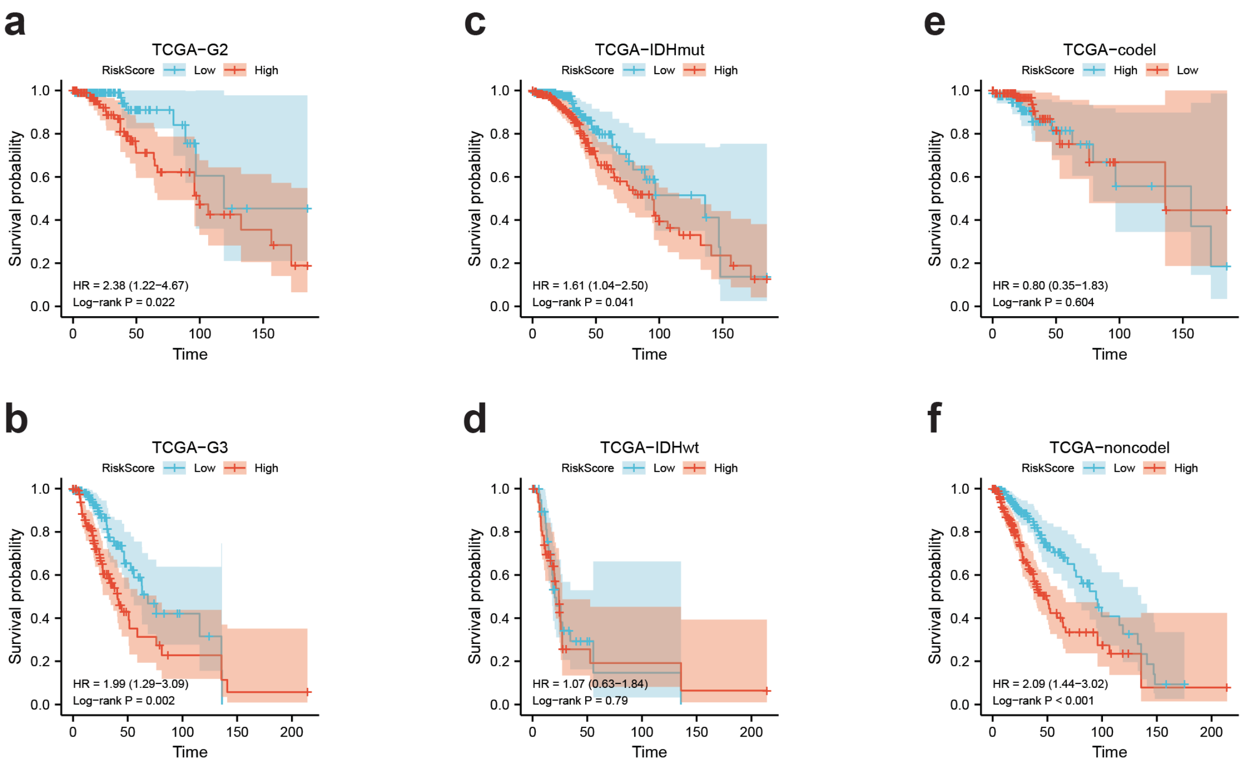

Figure S3: Kaplan–Meier curve of stratified analyses of the CRGs signature for associations with clinical characteristics in TCGA cohort, when dividing patients according to the subgroup median risk score. (a) OS curve in grade2 patients. (b) OS curve in grade3 patients. (c) OS curve in IDH mutate patients. (d) OS curve in IDH wildtype patients. (e) OS curve in 1q/19p codeletion patients. (f) OS curve in 1q/19p non-codeletion patients.
