# Supplementary material for: Cuproptosis-related gene signature stratifies lower-grade glioma patients and predicts immune characteristics
Source: Front Genet. 2022 Oct 25;13:1036460. doi: 10.3389/fgene.2022.1036460 (PMC9640744; doi:10.3389/fgene.2022.1036460)
Supplement: Supplementary file 5 [file Table2.DOCX]

| Characteristic | high | low | p |
| --- | --- | --- | --- |
| n | 46 | 126 |  |
| Gender, n (%) |  |  | 0.764 |
| Female | 19 (11%) | 47 (27.3%) |  |
| Male | 27 (15.7%) | 79 (45.9%) |  |
| Grade, n (%) |  |  | < 0.001 |
| G2 | 16 (9.3%) | 82 (47.7%) |  |
| G3 | 30 (17.4%) | 44 (25.6%) |  |
| IDH status, n (%) |  |  | < 0.001 |
| Mutant | 25 (14.6%) | 102 (59.6%) |  |
| WT | 21 (12.3%) | 23 (13.5%) |  |
| 1p/19q codeletion, n (%) |  |  | < 0.001 |
| codel | 0 (0%) | 55 (32.4%) |  |
| non-codel | 46 (27.1%) | 69 (40.6%) |  |
| Age, mean ± SD | 43.54 ± 13.54 | 39.32 ± 9.49 | 0.056 |

Table 2: Clinical characteristics of the LGG patients from the CGGA cohort
