# Supplementary material for: Cuproptosis-related gene signature stratifies lower-grade glioma patients and predicts immune characteristics
Source: Front Genet. 2022 Oct 25;13:1036460. doi: 10.3389/fgene.2022.1036460 (PMC9640744; doi:10.3389/fgene.2022.1036460)
Supplement: Supplementary file 6 [file DataSheet1.PDF]

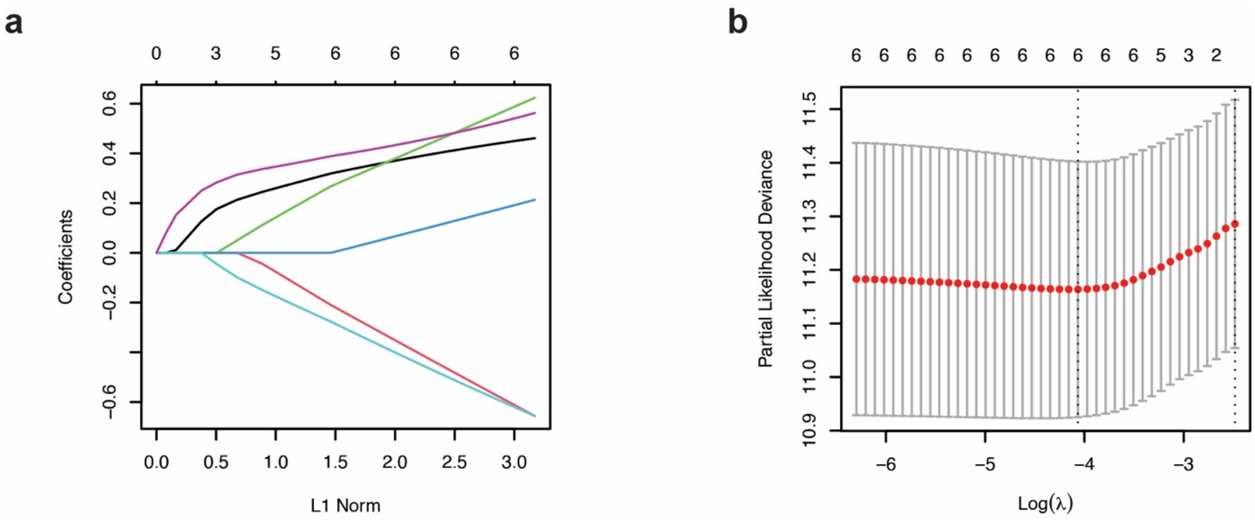

Fig. S1 Construction of a 6-gene signature model in the TCGA cohort. a. LASSO coefficient profiles of the expression of 6 candidate genes. b. Selection of the penalty parameter (λ) in the LASSO model via 10-fold cross-validation. The dotted vertical lines are plotted at the optimal values following the minimum criteria (left) and "one standard error" criteria (right).
