# Supplementary material for: Cuproptosis-related gene signature stratifies lower-grade glioma patients and predicts immune characteristics
Source: Front Genet. 2022 Oct 25;13:1036460. doi: 10.3389/fgene.2022.1036460 (PMC9640744; doi:10.3389/fgene.2022.1036460)
Supplement: Supplementary file 7 [file DataSheet5.PDF]

Editing Certificate

This document certifies that the manuscript

Cuproptosis-Related Gene Signature Stratifies Lower-Grade Glioma Patients and Predicts Immune Characteristics

prepared by the authors

Zihao Zhang, Bingcheng Wang, Xiaoqin Xu, Tao Xin

was edited for proper English language, grammar, punctuation, spelling, and overall style by one or more of the highly qualified native English speaking editors at AJE.

This certificate was issued on **September 28, 2022** and may be verified on the AJE website using the verification code **F04E-9533-4366-4D7A-15AP**.

Neither the research content nor the authors' intentions were altered in any way during the editing process. Documents receiving this certification should be English-ready for publication; however, the author has the ability to accept or reject our suggestions and changes. To verify the final AJE edited version, please visit our verification page at aje.com/certificate. If you have any questions or concerns about this edited document, please contact AJE at support@aje.com.

AJE provides a range of editing, translation, and manuscript services for researchers and publishers around the world.
For more information about our company, services, and partner discounts, please visit aje.com.
